# Resident and staff perspectives of person-centered climate in nursing homes: a cross-sectional study

**DOI:** 10.1186/s12877-019-1313-x

**Published:** 2019-10-29

**Authors:** Yunxia Yang, Hui Li, Lily Dongxia XIAO, Wenhui Zhang, Menghan Xia, Hui Feng

**Affiliations:** 1 Xiangya Nursing School, Central South University, 172 Tong Zi Po Road, Changsha, 410013 Hunan China; 2 Nursing Department, Ophthalmology Department, The third Xiangya Hospital of Central South University, Xiangya Nursing School of Central South University, 172 Tong Zi Po Road, Changsha, 410013 Hunan China; 3 College of Nursing & Health Sciences, Flinders University, GPO Box 2100, Adelaide, 5001 Australia; 4 School of Nursing and Department of Statistics & Data Sciences, The University of Texas at Austin, 1710 Red River Street, Austin, TX 78701 USA; 5 Health Nursing Research Center, Xiangya Nursing School of Central South University, 172 Tong Zi Po Road, Changsha, 410013 Hunan China

**Keywords:** Aged care, Nursing homes, Nursing staff, Older people, Person-centered care

## Abstract

**Background:**

Person-centered care is widely recognized as a gold standard and is based on a supportive psychosocial climate for both residents and staff in nursing homes. Residents and staff may have different perspectives as to whether the climate in which they interact is person-centered, perhaps due to their different expectations of the nursing home environment and the provision of care services. The aim of this study was to explore and compare resident and staff perspectives of person-centered climate in aged care nursing homes.

**Methods:**

This is a descriptive cross-sectional study using a cluster random sampling method. The study collected data in 2016 from residents (*n* = 251) and nursing staff (*n* = 249) in 23 nursing homes using a Person-centered Climate Questionnaire-Patient version and Person-centered Climate-Staff version. T-tests for independent-samples were used to compare scores ranked by nursing staff and residents.

**Results:**

The mean scores of ‘A climate of safety’ subscale and ‘A climate of everydayness’ subscale rated by residents were significantly lower than those rated by nursing staff. The mean scores of ‘A climate of hospitality’ rated by residents were very low among the three subscales, an indicator of the need to improve a more home-like environment for residents. Residents in larger size nursing homes showed a higher score of person-centered climate compared with their counterparts in small size nursing homes.

**Conclusions:**

This study reveals that the perspectives and perceptions of person-centered climate differ between residents and nursing staff. Therefore, both resident and staff perspectives should be taken into account in attempting to improve person-centered climate for better care outcomes.

## Background

Person-centered care is widely recognized as a gold standard and needs to be embedded into policies, regulations and quality improvement in aged care in a global context [1, 2]. This care approach emphasizes that each person is unique and worthy of respect regardless of disability or illness [3]. Person-centered care standards enable staff in aged care to achieve high-quality care for residents and improve resident satisfaction with care services [4–6]. This care approach is conditioned by a positive psychosocial environment in aged care homes where both residents and staff have a sense of being valued and receiving organizational support to achieve person-centered care [7–9]. However, research evidence in this field is mainly from high-income countries where the aged care system is better developed compared to the system in lower- and middle-income countries (LMICs) [10, 11]. Research evidence in person-centered care from LMICs is much needed considering that the most older people live in LMICs and the disparities of quality of aged care between in a global context [12]. Additionally, studies that compare resident and nursing staff perspectives of person-centered climate are scarce. This study addresses the gaps in research by engaging both residents and staff in assessing person-centered climate, from the perspective of a lower-middle income country (LMIC).

Unlike other LMICs, China shows the highest rate of population aging [13]. In 2015, the number of people aged 65 and over reached 144 million, which comprised 10.5% of the total population [14]. It is estimated that the proportion of older people will increase to 23% in 2050 [14]. Older Chinese are traditionally cared for by their family members due to the influence of filial piety [15, 16]. However, the number of family caregivers has decreased because of internal migration of the labor force, the tendency towards a small family size, and the “empty-nest families” [17, 18]. Consequently the growth of nursing homes has become significant in China in recent years [19]. It is expected that nursing homes will play a crucial role in the next few decades to meet the demand for caring for older people with complex care needs [13].

Nursing homes in China share many similarities with other LMICs where policies, resources and regulations are largely underdeveloped to govern quality care [20]. Although great efforts have been made to improve nursing home care by the Central Government in China, the workforce and the care environment to support high-quality care have become issues of concern [20–22]. Most staff employed in nursing homes are untrained workers with low education levels and the majority are migrants from rural areas with little experience in aged care [23]. It is predicted that the number of professional care workers in nursing homes in China will be 6.91 million in 2030 and 32.62 million in 2050 [19]. Research evidence on consumers’ expectations of person-centered high-quality care is much needed so as to inform education and training for staff.

High-quality care for residents in nursing homes has a core component of person-centered care [8, 24]. The concept of “person-centeredness” was first mentioned by Rogers [25] and was first applied to aged care by Kitwood (1997). Person-centered care refers to maintaining one’s personhood despite disabilities or illness, endeavoring to adopt the residents’ values and lifestyles, recognizing their life experiences and relationships, and creating a positive psychosocial environment for them to achieve the highest level of quality of life [1, 26, 27].

It has been strongly argued that person-centered care must be supported by a positive psychosocial environment in nursing homes [28]. Such an environment has the following characteristics: (i) valuing residents as individuals with unique life experiences; (ii) preserving their autonomy and dignity; (iii) encouraging them or their family members to participate in decision making involving their care, and (iv) organizational support for staff via education, adequate resources and staffing levels [29, 30]. Residents and staff may have different and sometimes competing views about what constitutes an ideal psychosocial environment due to their different interests and perspectives. For example, residents expect the nursing homes to be their own homes where they can exercise their autonomy to control the environment and the care activities to meet their care needs [31, 32]. Staff on the other hand, usually perceive the nursing home as a workplace where they can exercise their right to create a safe workplace, have a reasonable workload and in return receive a decent income [33, 34].

The concept of positive psycho-social environment was built into the Person-centered Climate questionnaire in a resident version and a staff version [8]. The Person-centered Climate Questionnaire-Patient version (PCQ-P) was originally developed in a hospital setting, but was validated and tested in nursing home settings [7, 35]. This scale comprises three subscales: a climate of safety, a climate of everydayness and a climate of hospitality [35]. A climate of safety focuses on a safe place for residents to be cared for while a climate of everydayness emphasizes an environment that enables residents to have daily positive experiences. A climate of hospitality represents residents’ expectations of being welcomed in a caring environment. The Staff version of PCQ shares similar subscales, namely a climate of safety, a climate of everydayness and a climate of community [26]. A climate of safety points out a safe workplace for staff while a climate of everydayness refers to staff expectations of an environment that enables them to have daily positive experiences. A climate of community represents staff expectations of a caring and welcome environment for residents while a positive psychosocial environment needs to be developed by both residents and staff together [8, 26]. Engaging both residents and staff in assessing the person-centered climate is the first step towards achieving an ideal climate expected by both residents and staff to support person-centered care.

## Methods

### Aims

The aim of the study was to explore and compare resident and staff perspectives of person-centered climate to facilitate both residents and staff engaged in person-centered care in nursing homes.

### Design

A cross-sectional survey was employed in the study.

### Participants

Participants recruited in the study were from nursing homes in Hunan Province, China where in 2015, the population in the province aged 60 and over was 11.64 million, and there were 326 nursing homes with a total of 275,000 beds [36]. A cluster random sampling method was used to recruit residents and staff from nursing homes in four geographic areas of the Province. The researchers searched the website of the Department of Civil Affairs of Hunan Province to identify a name list of all nursing homes in the province. SPSS version 23.0 was used to randomly select one city from each geographic area and then select two districts from the selected cities. The total number of nursing homes in these selected districts was 32. All nursing homes from these selected districts were invited to participate in the study via a letter of invitation sent to the facility manager.

In total 23 nursing homes, or 72% of the total numbers in the selected districts agreed to participate in the study, evidenced by returned letters and information about the total number of nursing staff and residents from nursing home managers. In total, there were 320 nursing staff who cared for approximately 2210 residents in these nursing homes. The size of nursing homes varied widely, ranging from those accommodating 36 residents to as many as 380 residents. The number of staff employed in these nursing homes ranged from 8 to 110. Regarding the facility ownership, there were 8 state-owned nursing homes and 15 not-for-profit privately-owned nursing homes.

All nursing staff, including registered nurses and personal care assistants, in the nursing homes were invited to participate to the study. Inclusion criteria for nursing staff were as follows: (1) having more than 1 month’s work experience in the nursing home; (2) ability to understand and complete the survey; (3) willingness to participate. Nursing staff who were on vacation, were not on duty and or who had engaged in advanced studies were excluded.

**Fig. 1:**
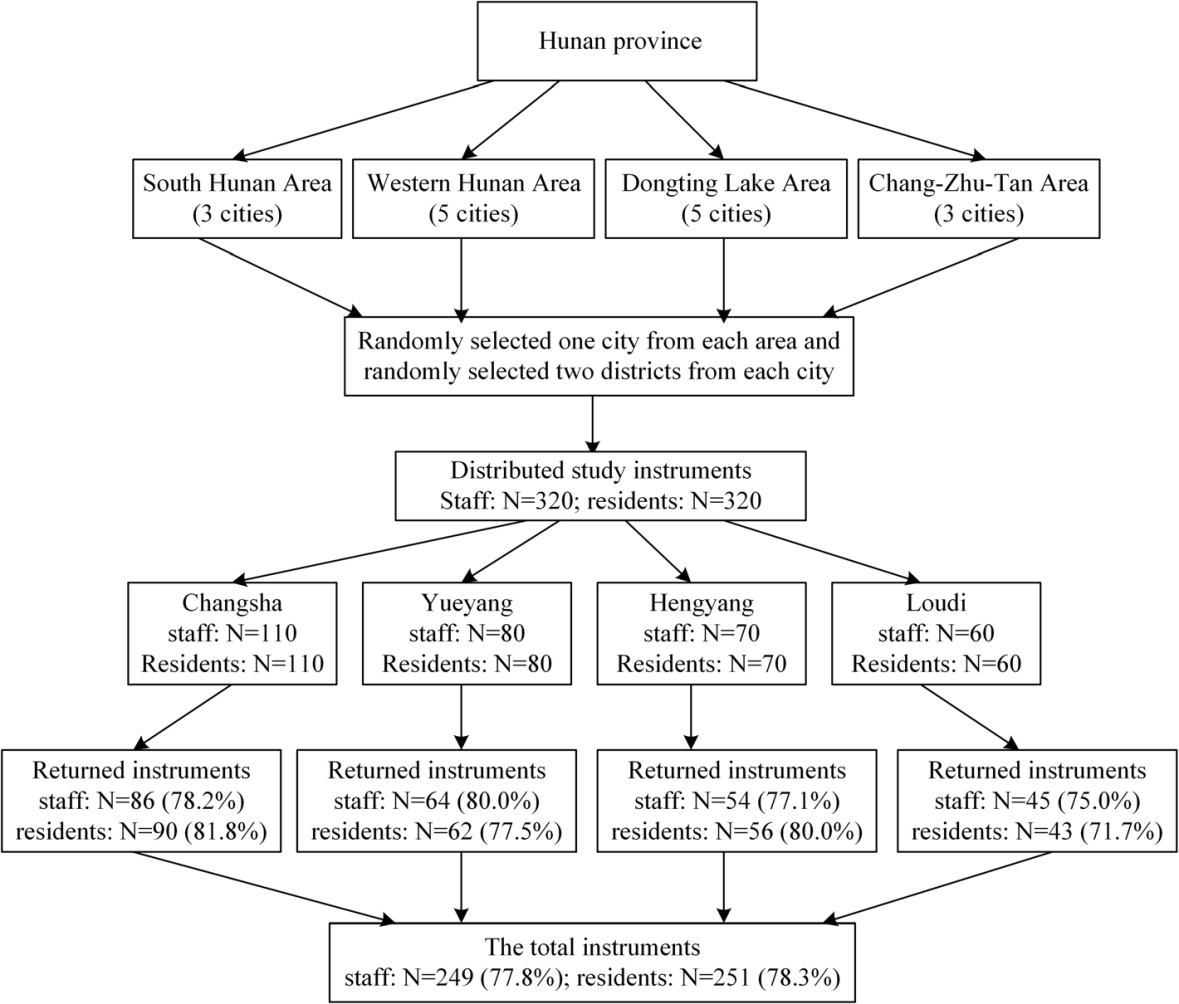
The sample frame

Residents were randomly selected to achieve a 1:1 resident to staff ratio if they met the following inclusion criteria: (1) aged 60 or over; (2) having lived in the nursing home for at least 1 month; (3) able to understand and respond to the questionnaires; and (4) willing to participate. The sample frame and numbers of participants from each city are shown in Fig. 1.

### Data collection

Data were collected from self-report questionnaires by staff and residents. A letter of invitation with the survey questionnaire was distributed to each potential participant in the nursing homes via their internal mail systems. Participants were assured that they could choose or refuse to participate in the survey without any consequences. Participants were provided with pre-addressed, prepaid and sealable envelopes to return their survey to the researcher directly. Residents were informed in the letter that researcher assistants would be available in their nursing homes and would visit them to help explain any questions they might have about the survey. All participants were reassured of strict confidentiality of the information they provided in the survey. This study was conducted from June 2016 to August 2016.

### Survey instruments

#### Person-centered climate questionnaire-patient

Resident perceived person-centered climate was assessed by the Person-centred Climate Questionnaire-Patient version (PCQ-P) [35]. The English version of the PCQ-P was validated in long-term care [7]. The scale is a 17-item scale with three dimensions: safety (10 items), everydayness (4 items), and hospitality or a caring and welcome place for residents (3 items). Responses ranged from 1 “completely disagree” to 6 “completely agree” with higher scores indicating a more person-centered climate.

#### The person-centred climate questionnaire-staff

The Person-centred Climate Questionnaire-Staff version (PCQ-S) [26] was used to measure staff perceived person-centered climate in their workplace. The PCQ-S contains 14 items and consists of three subscales: safety (5 items), everydayness (5 items) and community or a caring and welcome place for residents (4 items). A 6-point rating system is used and higher scores indicate a more person-centered climate.

Although the descriptive items of the three subscales in PCQ-P and PCQ-S slightly differ, they share the core concepts that the climate should be: (i) a safe place for residents and staff to interact with; (ii) a place for these two groups to have daily positive experiences; and (iii) a caring and welcome place for residents who are cared for in the environment. Additionally, PCQ-P and PCQ-S use the same 6-Likert scale scoring system rated from 1 to 6 with higher scores indicating more person-centered climate. Therefore, the similar factor (concept) structures and scoring systems of the PCQ-P and PCQ-S support the comparison of the three subscales from the two participant groups.

#### Physical self-maintenance scale

The Physical Self-Maintenance Scale (PSMS) questionnaire was used to measure the activities of daily living of residents [37]. Six basic functional abilities were assessed: toilet, feeding, dressing, grooming, physical ambulation and bathing. All items were rated by discussion between the resident and his/her caregiver on a 4-point rating scale from 1 (no impairment) to 4 (severe impairment). A total score of 6 is defined as activities of daily living intact and scores higher than 6 represent activities of daily living impairment. The PSMS has been widely used in China, and the PSMS for Chinese version has been shown to have satisfactory reliability and validity [38, 39].

#### Reliability and validity

As the Person-centered Climate Questionnaire-Staff version and Person-centered Climate Questionnaire-Patient version were originally developed in Swedish and no Chinese versions were available. The principal chief investigator contacted the original author of the scales and gained permission to translate and validate the two scales. A forward-backward translation method was used to translate the scales, and psychometric properties were evaluated. First, the research group translated the English versions into Chinese. Second, a professionally qualified translator carried out the back translation of the Chinese versions into English. Third, a thorough comparison between the original versions, the translated Chinese versions and the back-translated English versions was conducted to examine any discrepancies. Construct validity was estimated using a principal component analysis with varimax orthogonal rotation. The internal consistency reliability was evaluated using test-retest reliability and Cronbach’s α coefficient was calculated.

PCQ-P and PCQ-S had the same factor structure with original scales and most of the original items loaded on the same factor. The Chinese PCQ-S consists of 14 items and includes three factors: Factor 1-a climate of safety (3 items), Factor 2-a climate of everydayness (5 items) and Factor 3-a climate of community (6 items). The total Cronbach’s *α* coeffient for the Chinese PCQ-S was 0.91 and each subscale showed acceptable Cronbach’s α coefficient. The factor analysis result of the Chinese PCQ-S was summarized in Table 1. The Chinese PCQ-P consists of 17 items with three factors: Factor 1-a climate of safety (6 items), Factor 2-a climate of everydayness (7 items) and Factor 3-a climate of hospitality (4 items). The total Cronbach’s *α* coefficient for the Chinese PCQ-P was 0.93 and each subscale showed acceptable Cronbach’s α coefficient. The factor analysis result of the Chinese PCQ-S was presented in Table 2.

**Table 1:** Factor loading matrix of each item in Chinese PCQ-S ^a^(*n* = 249)

Items	Factor-1	Factor-2	Factor-3
	Safety	Everydayness	Community
S12 A place where it is easy for the residents to receive visitors			0.830
S11 A place where it is easy for the residents to keep in contact with their loved ones			0.830
S13 A place where it is easy for the residents to talk to the staff			0.797
S14 A place where the residents have someone to talk to if they so wish			0.637
S4 A place where the residents are in safe hands			0.482
S5 A place where the staff use a language that the residents can understand			0.447
S7 A place where there is something nice to look at		0.818	
S8 A place where it is quiet and peaceful		0.770	
S9 A place where it is possible to get unpleasant thoughts out of your head		0.720	
S6 A place which feels homely even though it is in an institution		0.666	
S10 A place which is neat and clean		0.613	
S1 where I feel welcome	0.822		
S2 where I feel acknowledged as a person	0.804		
S3 where I feel I can be myself	0.717		
Percent total variance explained(%)^b^	16.626	22.481	24.685

^a^*PCQ-S* Person-centred Climate Questionnaire-Staff version

^b^Bartlett’s test statistic was significant (χ^2^(91) = 2032.70, *p* < .05). Kaiser-Meyer-Olkin’s (KMO) indicated sampling adequacy appropriate for factor analysis (KMO = .90)

**Table 2:** Factor loading matrix of each item in Chinese PCQ-P ^a^(*n* = 251)

Items	Factor-1	Factor-2	Factor-3
	Safety	Everydayness	Hospitality
P9 A place which is neat and clean		0.741	
P14 A place where people talk about ordinary things, not just illness		0.737	
P4 A place where I feel welcome		0.684	
P11 A place where there is something nice to look at		0.648	
P3 A place where I feel in safe hands		0.648	
P13 A place where it is possible to get unpleasant thought out of your head		0.595	
P8 A place where the staff use language I can understand		0.570	
P15 A place where the staff make extra efforts on my behalf			0.885
P17 A place where I can get “that little bit extra”			0.875
P12 A place where which feels homely even though I am in an institution			0.662
P16 A place where I can make choices, for example what to wear			0.532
P6 A place where the staff take notice of what I say	0.811		
P5 A place where it is easy to talk to the staff	0.777		
P7 A place where the staff come quickly when I need help	0.664		
P1 A place where the staff is knowledgeable	0.565		
P2 A place where I rely on receiving the best care	0.548		
P10 A place where the staff have time for the residents	0.439		
Percent total variance explained (%)^b^	5.968	48.474	8.130

^a^*PCQ-P* Person-centred Climate Questionnaire-Patient version

^b^Bartlett’s test statistic was significant (χ^2^(136) = 2503.64, *p* < .05). Kaiser-Meyer-Olkin’s (KMO) indicated sampling adequacy appropriate for factor analysis (KMO = .93)

### Ethical considerations

The research was approved by the Nursing and Behavioral Medicine Institutional Review Boards, Xiangya School of Nursing, Central South University (Ref No. 2017035). Participating nursing homes accepted the ethical approval. An information pack was provided for participants that explained the purpose of the study, participants’ right to refuse to participate in the study, the benefits and potential risks, and strategies used to ensure the confidentiality of information that participants provided. An information session was also provided for staff and residents to ask questions they might have in relation to their participation in the study. The survey utilized anonymity and participants were not asked to provide their identity. Each nursing home site was assigned a code for data comparison purposes. Returning a completed survey to the researchers was regarded as evidence of participants’ consent to participate in the survey.

### Data analysis

SPSS version 23.0 was used for data analyses. Descriptive statistics were used to summarize data. Subscale means, standard deviations (SD), 95% confidence interval of subscale means, percentages and frequencies were calculated for items and scales. Internal consistency for the PCQ-P and PCQ-S was estimated by calculating Cronbach’s *α* coefficents, and factor structure was evaluated by exploratory factor analysis. T-tests for independent-samples were used to compare scores ranked by nursing staff and residents on the three sub-scales of person-centered climate. Values of *p* < 0.05 was considered significant statistics for all analysis.

## Results

The demographic characteristics of staff were presented in Table 3. Staff had an average age of 47.8 years old and had an average of a 2.1-year experience in nursing home care. The majority of staff were female (90.8%), married (79.1%) and had junior middle school education or below (62.3%). Most of the staff participants were personal care assistants (79.5%) and Registered Nurses made up 20.5% of staff participants.

**Table 3:** Characteristics of staff participants (*n* = 249)

Variable	n (%)	Mean (SD)
Gender		
Male	23 (9.2)	
Female	226 (90.8)	
Age (years)		47.8 (11.9)
Marital status		
Unmarried	31 (12.4%)	
Married	197 (79.1%)	
Divorced or widowed	21 (8.5%)	
Education		
Primary school or below	39 (15.7%)	
Junior middle school	116 (46.6%)	
Senior high school	78 (31.3%)	
Bachelor or master	16 (6.4%)	
Years of experience in facility		2.1 (1.9)
Qualifications		
Registered nurses	51 (20.5%)	
Nurses’ assistants	198 (79.5%)	
Attending training activities in aged care		
Yes	226 (90.8)	
No	23 (9.2)	

The demographic characteristics of residents were summarized in Table 4. Resident participants had a mean age of 82.3 years old and the mean length of stay in the nursing homes was 14 months. Most residents were female (59.8%) and more than half of residents (50.2%) had activities of daily living impairment. The majority of residents (63.8%) also had two or above chronic diseases.

**Table 4:** Characteristics of resident participants (*n* = 251)

Variable	n (%)	Mean (SD)
Gender		
Male	101 (40.2%)	
Female	150 (59.8%)	
Age (years)		82.3 (10.4)
Marital status		
Unmarried	9 (3.6%)	
Married	75 (29.9%)	
Divorced or widowed	167 (66.5%)	
Education		
Primary school	83 (33.1%)	
Junior middle school	71 (28.3%)	
Senior high school	67 (26.7%)	
Bachelor or master	30 (12.0%)	
Length of stay in facility, months		14 (7.9)
BADL ^a^		8.81 (4.10)
impaired	126 (50.2%)	
intact	125 (49.8%)	
The number of chronic diseases ^b^		
None	29 (11.6%)	
One	62 (24.7%)	
Two	84 (33.5%)	
Three or above	76 (30.3%)	

^a^*BADL* Basic Activities of Daily Living

^b^Based on International Classification of Diseases -10th version and categories used in previous studies, chronic diseases identified in present study included hypertension, stroke, diabetes, heart disease, bronchitis, pneumonia, tumor, et al [40, 41]

### Perceived person-centered climate

In the PCQ-P, the mean total score was 59.7 (SD 11.5). The subscale of a climate of hospitality was scored the lowest (mean 2.72, SD 0.78), followed by a climate of safety subscale (mean 3.25, SD 0.76) and a climate of everydayness (mean 4.19, SD 0.74). The result revealed that residents had a relatively low level of sense of being welcomed in the care home.

In PCQ-S, the mean total score was 52.1(SD 9.4). The subscale that showed the lowest mean-score was the climate of everydayness (mean 4.49, SD 0.86), followed by a climate of safety (mean 4.55, SD 0.82) and a climate of community (mean 5.01, SD 0.71). The result indicated that staff had some level of agreement that the environment would enable them to have positive experiences and a feeling of safe workplace. Staff indicated strongly agreed that the nursing home was a caring community for residents.

### Comparisons of mean subscale scores of PCQ-P and PCQ-S

Comparison of mean subscale scores of PCQ-P and PCQ-S revealed that staff perceived significantly higher mean scores for subscales of (i) safety [Mean difference 1.30 (95% CI 1.16–1.44), *p* < .001], (ii) everydayness [Mean difference 0.30 (95% CI 0.16–0.45), *p* < .001] and (iii) a caring and welcome climate [Mean difference 2.28 (95% CI 2.15–2.41), *p* < .001] (see Table 5).

**Table 5:** Comparisons of subscale scores between staff and residents

Subscales	Staff (*n* = 249)	Residents (*n* = 251)	Mean differences	Group comparison
	Mean (SD ^a^)	Mean (SD)	Mean (95% CI ^b^)	*p* value
Safety	4.55 (0.82)	3.25 (0.76)	1.30 (1.16, 1.44)	*p* < 0.001
Everydayness	4.49 (0.86)	4.19 (0.74)	0.30 (0.16, 0.45)	*p* < 0.001
Community/Hospitality	5.01 (0.71)	2.72 (0.78)	2.28 (2.15, 2.41)	*p* < 0.001

^a^*SD* standard deviation

^b^*CI* Confidence interval

### Differences based on the size of nursing homes

When comparing smaller nursing homes (≤60 residents) with larger nursing homes (> 60 residents), there were significant differences in the subscales ‘a climate of safety’ (*p* = .048) and ‘a climate of everydayness’ (*p* < .001) for residents. Residents in larger nursing homes perceived better climate of person-centeredness, a better climate of safety and everydayness than those in smaller facilities (see Table 6). There were no significant differences on the ratings of nursing staff between the large and smaller nursing homes (*p* > 0.05).

**Table 6:** Impacts of facility size on participants’ person-centeredness

Subscales	Larger facilities (> 60 residents)	Smaller facilities (< 60 residents)	Mean differences	Group comparison
	Mean (SD)	Mean (SD ^a^)	Mean (95% CI ^b^)	*p* value
Safety- PCQ-S ^c^	4.59 (0.85)	4.38 (0.65)	0.22 (0.00,0.44)	*p* = 0.053
Everydayness- PCQ-S	4.51 (0.85)	4.40 (0.93)	0.11(−0.17,0.38)	*p* = 0.437
Community- PCQ-S	5.00 (0.70)	5.04 (0.77)	−0.04(−0.27,0.18)	*p* = 0.723
Safety- PCQ-P ^d^	3.29 (0.71)	2.89 (1.04)	0.40 (0.08,0.71)	*p* = 0.013
Everydayness- PCQ-P	4.25 (0.71)	3.66 (0.80)	0.58 (0.28,0.88)	*p* < 0.001
Hospitality- PCQ-P	2.73 (0.76)	2.64 (0.95)	0.09(−0.23,042)	*p* = 0.579

^a^*SD* Standard deviation

^b^*CI* Confidence interval

^c^*PCQ-S* Person-centred Climate Questionnaire-Staff version

^d^*PCQ-P* Person-centred Climate Questionnaire-Patient version

### Differences based on the ownership of nursing homes

When comparing state-owned nursing homes and not-for-profit privately-owned nursing homes, there were no significant differences in any subscales (*p* > 0.05).

## Discussion

The present study compared resident and staff perspectives of person-centered climate in nursing homes and identified differences that would provide a better understanding of residents’ expectations of a positive climate to support person-centered care. Findings indicated that residents perceived a lower level of person-centered climate in the nursing homes in which they lived compared with staff. Residents in larger nursing homes perceived better climate of person-centered care compared with their counterparts in smaller facilities.

In the present study, the total mean score of PCQ-P was 59.7 (SD 11.5) and was much lower than the total mean score of 86.5 (SD 11.4) reported in a study in Norway [9]. The gap reveals the room for improvement person-centered care for residents in nursing homes in China. The gap also supports the need to conduct studies in this field across the global to promote best practice. Person-centered care ranked by residents is viewed as a crucial quality indicator [5]. A low score should trigger internal investigation into the quality of care for residents by the management group in nursing homes. It is widely recognized that creating person-centered climate requires a system approach to ensure governance, regulation, staffing level and educated preparation for staff to support person-centered care [2, 5]. A low score of person-centered care perceived by residents should also be an indicator for policy review and resource development in the aged care system.

In this study, the perception of person-centered climate by staff was 52.1(SD 9.4) and was lower than the total mean score of 69.9 (SD 9.0) reported in Swedish nursing homes, using the same tools (PCQ-S) [8, 28]. The workload and working conditions across countries might have contributed to the different perspectives of staff about person-centered climate. Nursing homes in China experienced staff high turnover intention, poor staffing stability and found it difficult to retain staff with quality and experience in aged care [20, 42]. These workplace issues were reported as the main factors affecting quality of care, lack of a person-centered care approach for residents and lower staff job satisfaction. Improving staff perspectives of person-centered climate requires organizational cultural change and policy development to value care staff and their contributions to the aged care industry and support them to develop their careers in aged care.

In the present study, the high mean scores (‘somewhat agree’) from staff and the low mean scores (‘somewhat disagree’) from residents on safety climate provided evidence that residents’ expectation of a safe climate was much higher for them than for staff. A climate of safety from the residents’ perspective is usually related to an environment where staff are approachable, available for residents and are competent to care for them [26]. It has been well-documented that safety climate of nursing homes was associated with staffing levels, staff workload and their abilities to identify and meet residents’ care needs in a timely manner [2, 5]. Although the shortage of staff and a low level of staffing were widely reported in higher-income countries, these situations seemed to be much worse in China due to lack of national standards and regulation for staffing levels, and education and training requirements for staff in nursing homes [20, 42]. It has been reported that most nursing homes in China provided residents with very basic services including personal care, basic medical care, room cleaning, meals and laundry, and that the standards and the quality of these care services were not monitored and regulated [42]. Personal care assistants were mainly untrained and had heavy workloads that might have contributed to their inability to attend and respond to residents’ care needs in a timely manner in nursing homes in China [20, 42]. A quality improvement process should address residents’ expectations of a safe environment.

Everydayness climate indicates that an environment enables positive experiences and the sense of belonging to the adopted home as perceived by residents [35]. The higher scores by staff and the lower scores by residents on everydayness climate provided evidence that residents did not share the same level or sense of positive experiences with staff. Studies identified that factors affecting residents’ sense of positive experiences included their autonomy to control their lives, their preferences, care plans and treatment in the nursing home [31, 32]. In these studies, residents usually had a sense of loss of their own homes and had negative experiences when they were admitted to the nursing homes. However, studies also identified that residents were capable of adjusting to their new life in nursing homes if the climate of the nursing homes supported their transition from home care to nursing home care [43]. Developing relationships with staff and other residents were indicators of their successful transition [31, 43]. In China, most personal care assistants were untrained, migrant workers from rural areas who were lacking professional skills necessary to support residents to exercise their autonomy, their preferences and to build meaningful relationships with others [23].

The finding supported previous studies that residents in larger nursing homes perceived a higher level of person-centered climate in the total scale, the subscales of ‘a climate of safety’ and ‘a climate of everydayness’ than those in smaller nursing homes [9]. In the previous study, the differences were explained in that large nursing homes created more spaces for residents to spend the day in different areas of the facility, and had more opportunities to socialize and interact with other residents [9]. Additionally, large nursing homes may also attract more competent employees. Therefore, residents may have had positive experiences and developed good relationships with staff whereby they developed a sense of home and sense of safety.

### Limitations

There are several limitations of this study. First, the survey was based on self-selection to participate. The findings therefore might not represent the perspectives of those who chose not to participate in the study. Second, the nature of self-administered survey is associated with bias. Participants, particularly staff might have chosen socially accepted answers in responding to the questions. Third, as this study was undertaken in one province in China, the findings may not reflect nursing homes located in other provinces or regions. In addition, most participants in the study had no cognitive impairment and therefore the findings cannot be generalized to these with cognitive impairment in nursing homes.

## Conclusion

In a country with an underdeveloped residential aged care system, residents and staff rank person-centered climate of nursing homes relatively low compared with their counterparts in countries with well-established residential aged care systems. The levels of person-centered climate perceived by residents are significantly lower than that perceived by nursing staff. The findings also indicate that large nursing homes seem to demonstrate better person-centered climate and further studies are needed to explore the factors contributing to these outcomes. The lack of regulations on staffing, workload and staff education and training in China might have contributed to the situations. In addition, the lack of culture to support consumer-directed care in nursing homes in China might also have contributed to the low scores of Person-centered Climate Questionnaire in residents. More studies are needed to explore the impact of these factors on the quality of care for residents. As person-centered climate is one of quality indicators in nursing home care, the findings suggest that both residents and staff need to be engaged in assessing this indicator to identify room for quality improvement. The PCQ-P and PCQ-S provide nurses in leadership and management positions with useful instruments to explore and engage both residents and staff to assess person-centered climate. These tools can be used on items, subscales or total scales based on the need to improve the quality of care for residents or for staff development purposes. Nursing managers should pay attention to the different mean subscale scores from residents and staff, to investigate factors that contributed to the differences. They can also use the results of the mean scores from the three subscales to plan staff development sessions to improve staff knowledge, skills and competence in providing residents with person-centered high-quality care. Nursing managers should also use the assessment outcomes to plan actions to improve organizational culture to facilitate and provide adequate staffing levels and support for staff development.

## Data Availability

The datasets generated and/or analysed during the current study are available from the corresponding author on reasonable request.

## Abbreviations

PCQ-P: Person-centred Climate Questionnaire-Patient version
PCQ-S: Person-centred Climate Questionnaire-Staff version
PSMS: Physical Self-Maintenance Scale
